# Heavy Metals Levels in Soil, Water and Feed and Relation to Slaughtered Camels’ Tissues (*Camelus dromedarius*) from Five Districts in Saudi Arabia during Spring

**DOI:** 10.3390/life13030732

**Published:** 2023-03-08

**Authors:** Mutassim M. Abdelrahman, Ibrahim A. Alhidary, Abdulkareem M. Matar, Mohsen M. Alobre, Moez Ayadi, Riyadh S. Aljumaah

**Affiliations:** 1 Department of Animal Production, College of Food and Agriculture Sciences, King Saud University, Riyadh 11451, Saudi Arabia; 2 Département de Biotechnologie Animale, Institut Supérieur de Biotechnologie de Beja, Université de Jendouba, Av. Habib Bourguiba, Beja 9000, Tunisia

**Keywords:** camels, heavy metals, tissues, feed, soil, water, districts, ICP-OES

## Abstract

Soil, water, and forage contaminated with toxic heavy metals such as Pb, Cd, and Co can affect the meat and liver quality of camels raised in this area which affect human health. This study aimed to determine the concentrations of Pb, Cd and Co in soil, water, feed and young camels’ carcass tissues (meat, liver, whole blood, rumen fluid and rumen tissue) from five districts in Saudi Arabia during the spring. All samples were wet-digested and analyzed by ICP-OES for heavy metals. In the liver, a significantly higher (*p* < 0.05) concentration of Pb and Co was observed in camels reared in the central and southern districts, while Cd was found significantly higher in the western and northern districts. The concentration of Pb, Cd and Co in meat of camels in the northern district was significantly higher (*p* < 0.05), and the meat of camels in the west had a higher (*p* < 0.05) concentration of Co. In addition, the Cd and Co concentrations in rumen fluid samples from camels in the eastern district were significantly higher than in the central district. A negative correlation between the concentration of Pb and Cd in rumen fluid and rumen tissue was reported. The accumulation of Cd, Pb, and Co in meat and liver was below the recommended maximum limit. Therefore, the harmful risk of human consumption of camel meat and liver is not possible.

## 1. Introduction

Heavy metals and semimetals are pollutants that pose a major potential environmental and health threat worldwide [1]. Heavy metals are chemical elements with a high density (greater than 4 g/cm^3^), mass, and atomic weight above 20; they are toxic at low concentrations. Some of these elements include aluminum (Al), beryllium (Be), copper (Cu), iron (Fe), manganese (Mn), Cd, mercury (Hg), and lead (Pb) [2]. 

One of the biggest problems at the environmental level at present is the contamination of the world’s water sources, soil and plant resources by heavy metals, as these are considered a serious problem for the global population [3,4]. The concentration of these metals is due to various anthropogenic activities that also increase the potentially harmful impacts on various ecosystems and the environment, causing serious problems at the economic level both locally and nationally [5].

Heavy metal toxicity is one of the main causes and threats to environmental health and is potentially dangerous due to their entrance to the food chain and the uptake of these metals by humans and other organisms [6,7]. These toxic elements have unknown biological functions and can have adverse effects on humans by damaging body organs [8] where they exert their toxicity by competing with essential metals at the sites of active enzymes or membrane proteins [9]. They are toxic to living organisms when their concentration exceeds the tolerable limit of the cells in the body [6].

A major route of exposure to Cd and Pb is through feed, which could be contaminated with metals from various sources such as air, water, and soil [10]. Several studies have shown that heavy metals such as Cd, and Pb have the ability to accumulate in the muscles of cows, camels, and sheep, but at lower concentrations than those found in kidney and liver [11,12]. Over time, heavy metals, even at low concentrations, can accumulate in the body and become toxic or carcinogenic and become the most harmful elements to animals and humans [13]. As a result of animals grazing on contaminated land, their carcass’s organs contain higher levels of heavy metals [14].

Meat and liver are important sources of trace elements such as Fe, Cu, Zn, and Se in human nutrition, but they may also contain toxic metals as residues, which have negative effects on human health and give a good indication of environmental pollution such as the contamination of feed, soil and drinking water [15]. 

The camel (*Camelus dromedarius*) is the most common ruminant species exposed to an environment with all sources of heavy metal pollution. Living in a harsh environment in arid and semi-arid areas, this animal has high production and reproductive efficiency [16]. Because of this, camels are a perfect source of meat and milk, especially in areas where the climate affects the performance of other farm animals [17]. Due to the high capacity of camels and differences in the structure of the digestive system, camels can tolerate toxic dietary components, such as heavy metals, compared to other ruminants. 

At present, there are environmental pollutants from various sources such as waste, fertilizers, oil refineries, and mining, which negatively affect the soil, water, vegetation and air [18]. Districts differ from each other depending on the variation of environmental parameters. They are affected by the seasons, which significantly affect the vegetation and pollution, and therefore the quality and quantity of natural pastures.

This study aims to determine the concentration of Pb, Cd, and Co in water, soil, and plant forage and their toxicity in camel’s meat, liver, whole blood, and rumen fluid and tissues. In addition, to obtain the baseline of absorbance of these metals by their solubility in rumen fluid and accumulation in rumen tissues, and to determine the correlation between these metals in different tissues.

## 2. Materials and Methods

### 2.1. Districts and Temperatures

**Figure 1:**
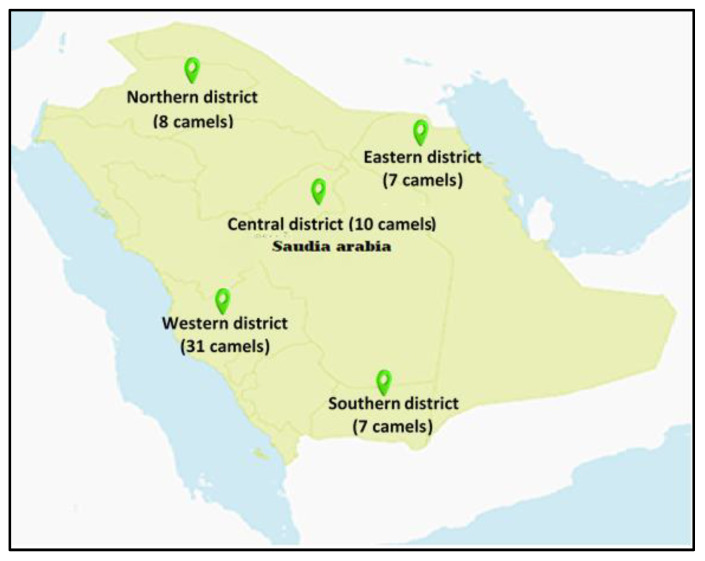
District distribution in Saudi Arabia for camels’ samples collection during spring season.

This study was conducted in five selected districts in Saudi Arabia during spring season and collected 177 samples divided by district (Figure 1): A. Central district (Riyadh: 50 samples, 10 camels); B. Western district (Mecca: 62 samples, 31 camels); D. Eastern district (Dammam: 35 samples, 7 camels); C. Southern district (Najran: 14 samples, 7 camels); and Northern district (Al-Jouf: 16 samples, 8 camels).

**Figure 2:**
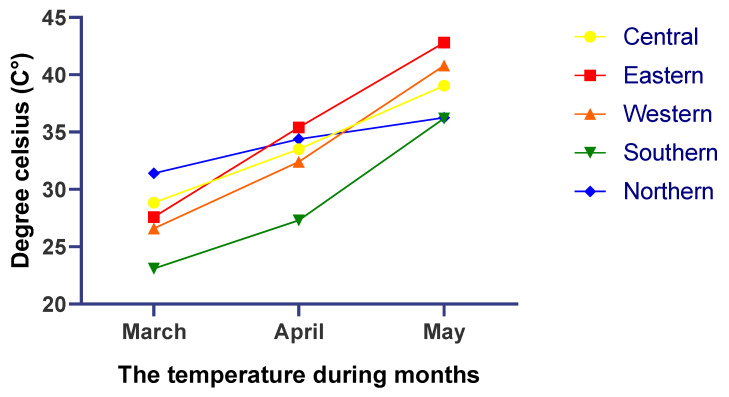
Temperature (°C) in different districts during spring season.

Temperature gradually increase in all districts from March to April (Figure 2). For each district, five representative samples of soil were collected from a depth of 100 cm. Drinking water samples were also collected from the same district. Representative soil and water samples were taken to be analyzed for heavy metals concentration besides the feed. Samples were drawn into a sterilized container. In general, grazing plants are available during the spring season, so alfalfa hay and Rhodes grass supplementation were reduced in all districts.

### 2.2. Samples Collection and Preparation

Only male camels ≥ one year old were selected from each district slaughterhouses to be as homogeneous as possible for sample collection. The samples were obtained from the five districts as follow: whole blood (17 samples), liver (63 samples), meat (63 samples), and rumen fluid (17 samples) and tissues (17 samples). Rumen fluid and tissues and whole blood were collected from only two districts (central and eastern districts). All fluid samples and tissues were collected by a veterinary specialist and immediately packed in labeled plastic bags, then transported to the laboratory in an Ace-Box to a cooler and stored at −20 °C until analysis

For sample preparation, all samples were wet-digested using acids (AOAC, 1995) and analyzed for Pb, Cd and Co concentration using ICP-OES according to Abdelrahman et al. [19] and Harris et al. [20]. Briefly, the whole blood, rumen fluid and tissues, liver and meat were prepared as follows: 0.50 ± 0.001 g tissue samples, liver, meat, and rumen tissues were weighed and placed in digestion flasks. One ml of the rumen fluid and whole blood was also placed in the digestion flask for acid digestion. Then, 3 mL of HNO_3_ (65%), 1 mL of HCl (36%), 1 mL of H_2_O_2_ (30% *w*/*v*) and 1 mL of deionized H_2_O were added to the sample before loading it on the digestion units. The samples were digested according to the AOAC (1990) recommendation. The digested samples, clear fluids, were diluted in a 25 mL volumetric flask using 0.1 normality HCl and mixed very well for subsampling in sterilized tubes (5 mL). 

### 2.3. Heavy Metals Analysis

The heavy metals (Cd, Co and Pb) were determined by using ICP-OES equipped with a Meinhard Nebulizer type A^2^. Argon (purity higher than 99.999%) supplied by AH group was used to sustain plasma and as carrier gas. The operating conditions for the ICP-OES minerals determination were as follow: 1300 W RF power, 15 L min^−1^ plasma flow, 0.2 L min^−1^ auxiliary flow, 0.8 L min^−1^ nebulizer flow, 1.5 mL min^−1^ sample uptake rate. Axial and radial views were used for metals determination, while 2-point background correction and 3 replicates were used to measure the analytical signal with the processing mode being the peak area. The emission intensities were obtained for the most sensitive lines free of spectral interference. The calibration standards were prepared by diluting the stock multi-elemental standard solution (1000 mg/L) in 0.5% (*v/v*) nitric acid. The range of the calibration curves for all minerals was from 1.0 ng mL^−1^ to 1.0 µg mL^−1^ (1–1000 ppb). The above procedure was also followed for the soil, feed and water samples to determine Cd, Pb and Co concentrations.

### 2.4. Statistical Analysis

The complete randomized design model was used to analyze the data using ANOVA (variance of analysis). The Proc GLM procedure of the SAS software (v. 9.4, SAS Institute Inc., Cary, NC, USA) was used to analyze the variables. The concentration of the heavy metals Cd, Pb, and Co was considered as a dependent variable and the five districts as independent variables. The relationship between the camels’ tissues and the heavy metals Cd, Pb, and Co was assessed using the Pearson correlation test. Mean differences were considered significant when the *p* value was ≤0.05.

## 3. Results

The current study investigates the levels of Pb, Cd and Co in camel tissues (liver, meat, whole blood and rumen fluid) as indication of environmental pollution, as well as the mechanism of heavy metal absorption.

**Figure 3:**
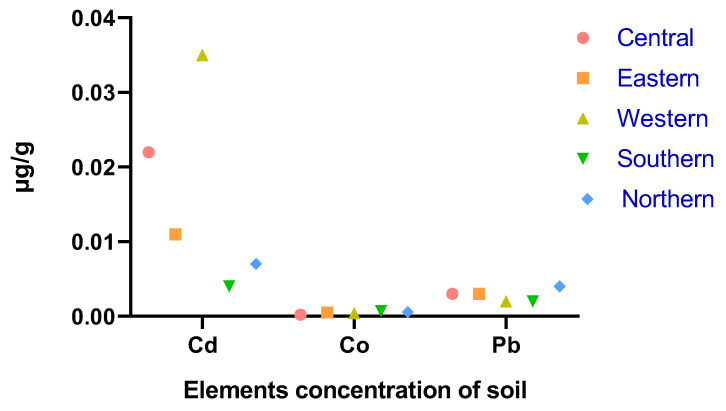
Heavy metals levels in soil samples collected from the five districts during spring.

**Figure 4:**
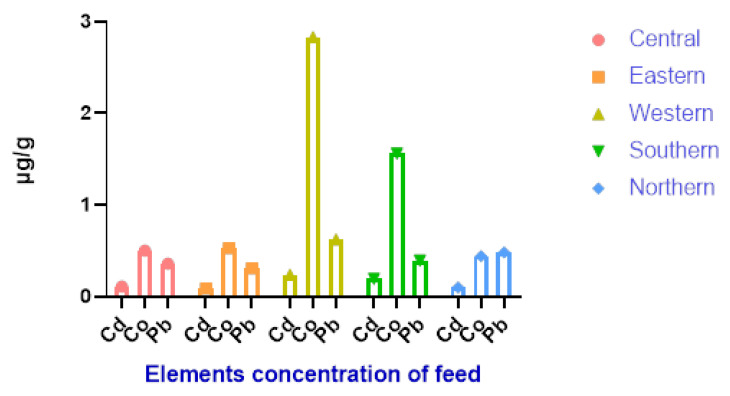
Heavy metals levels in feed samples collected from five districts during spring.

**Figure 5:**
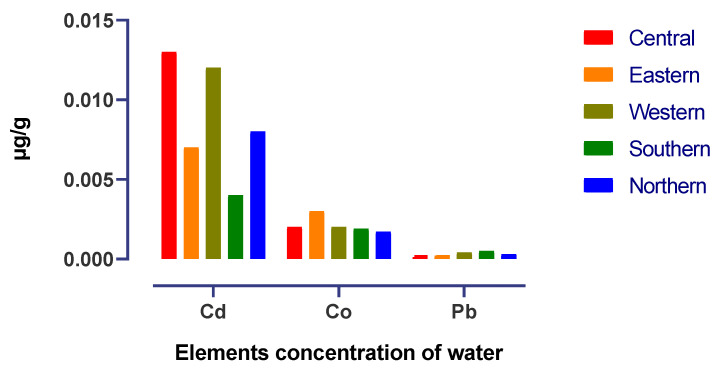
Heavy metals levels in water samples collected from the five districts during spring.

Figure 3 shows that the maximum soil Cd concentration values were observed in the western and central districts while the minimum values were observed in the southern district, and Pb and Co were found in the same range in all districts. As shown in Figure 4, the mean accumulation of Co in the feed in the western and southern districts was higher than that of other heavy metals in all districts. In contrast, Figure 5 shows that the content of Cd in the water was higher in the central and western districts than in other districts.

The results showed a significantly (*p* < 0.001) higher concentration of Pb in camel liver samples from the central district compared to other districts, followed by camel liver samples collected in the northern and eastern districts while the lowest concentration of Pb was observed in camel livers from the western and southern districts as showed in Table 1. The trend differed for Cd, which was significantly (*p* < 0.001) higher in concentration in the livers of camels from the western district compared to other districts. On the other hand, the concentration of the element Co was significantly (*p* < 0.001) higher in the livers of camels living in the central and southern districts, followed by livers of camels from the west, and the lowest value was observed in the livers of camels from the eastern and northern districts (Table 1).

Heavy metal concentrations in camel meat are shown in Table 2. The Pb level was significantly (*p* < 0.01) higher in Saudi Arabian camels reared in the northern district, while the Cd level was significantly (*p* < 0.01) higher in camels from the central and northern districts. In contrast, the Co levels were significantly higher in meat samples from northern and western camels compared to other samples.

Table 3 shows that the whole blood from camels reared in the central district had a higher mean value of Co accumulation (0.504 vs. 0.0379 µg/mL) compared to camels from the eastern district. No significant statistical difference was found between Pb and Cd accumulation in the whole blood in the two districts. In addition, the Cd and Co concentrations in rumen fluid samples were significantly (*p* < 0.05) higher in camels from the eastern district than in those from the central district (0.022 vs. 0.017 µg/mL; 0.11 vs. 0.063 µg/mL) whereas the Pb concentration in the rumen fluid did not differ from district to district (Table 3). On the other hand, the Co level in the rumen tissue was significantly (*p* < 0.05) higher in camels reared in the eastern district compared to camels reared in the central district (0.518 vs. 0.362 µg/mL), but the Cd and Co values were numerically higher in the camels from the eastern district compared to camels from the central district as illustrated at Table 3.

A significant negative correlation coefficient was reported between Pb and Cd in rumen fluid and rumen tissue (r = −0.954 and r = −0.981, respectively) as shown in Table 4 and Table 5. While Co showed no correlation with various camel tissues as shown in Table 6. Interestingly, the other tissues (meat and liver) did not appear to correlate with heavy metals in the rumen fluid, which has high concentrations of these elements.

## 4. Discussion

The main concern of dietary heavy metals is their accumulation in animal tissues (meat and liver), which can cause risks to human and animal health. In ruminants, the rumen can be the most important site for generation of microbial fermentation products. The uptake of a substance by the papillae depends on the mode of transport and the uptake capacity. The soluble minerals in the rumen fluid can bind to microbes, feed particles and fermentation metabolites. 

Camels have the ability to adapt to harsh environments, poor quality forage, and scarcity of water. This leads to large differences in the digestive system, especially in the forestomach, compared to other ruminants, resulting in camels potentially having a high ability to tolerate toxic dietary components, including toxic heavy metals [19]. In addition, heavy metal levels in animal tissues are mainly dependent on environmental pollution, rangeland, water, soil, and oil refinery waste [21]. 

The heavy metal levels in soil and water in the current study were different for each district (Figure 3 and Figure 5), with lower levels reported for most of them in the southern and northern parts of Saudi Arabia. This can impact the regional mineral status of animals grazing in these different areas. The differences in this reported prevalence may be due to mining activities, household waste, and other industrial pollution sources contaminating the environment in different geographic areas. Soil, water, and forage are the main sources of dietary minerals for camels reared in semi-arid environments. However, in spring, due to the availability of cover plants in all districts, dietary regimes were based on browsing and reduced supplementation compared to summer and winter [19]. 

In the current study, the liver Pb concentration in camels from the central district was 2.98 µg/g, higher than in other districts. This value was lower than that reported by Abdelbasset et al. [22] (1.33 mg/g) in livers of camels from Morocco, and higher than that reported by Bala et al. [12] (0.35–1.17 mg/kg) in livers of camels from Nigeria. In contrast, the Pb concentration in livers of camels from Saudi Arabia was lower than that in camels from Kirkuk (5.764 ppm) [23]. Furthermore, the Pb concentration in the liver of camels was higher than the Pb result in the livers of camels of different breeds such as Wadha, Maghateer and Magaheem (0.142, 0.204 and 0.179 mg/kg, respectively) in Saudi Arabia [24]. In addition, the Pb concentration in the liver of camels was higher than that of bovine (1.17 mg/kg) and sheep (1.17 mg/kg) [22].

The results obtained showed that the Pb concentration in camel meat from all districts was higher (between 1.001 to 2.058 µg/g) than reported by Abdou and Mohamed [25], who reported that the Pb concentration was 0.11 µg/g and then the muscle (0.11–0.20 mg/kg) from camels Nigeria [12]. Also lower than camel meat (2.01 mg/kg) in Algeria [11]. In contrast, in Saudi Arabia, Alturiqi and Albedair [26] reported that the lowest value for camel meat was recorded in the northern district at 2.01 µg/g and the highest value in the eastern district at 5.48 µg/g. In this study, the Pb level in liver and meat of camels from the central and other districts is below the allowable limit recommended by the European Commission (0.5 mg/kg) (EFSA) [27].

According to Figure 4, a higher Co content in the feed intake could explain the increased Co concentration in the livers of camels reared in the southern district. This gives an indication of the pollution in this district. The Co levels found in the liver samples from the central district were higher than those reported by Ibrahim et al. [3] (1.87 ± 0.35 ppm) and Bakhiet et al. [28] (2.20 mg/g), but they were within the range given by Ibrahim et al. [3] and Bakhiet et al. [28] for liver samples from the eastern district. In contrast, the Co concentration in liver samples from the western districts (2.461 µg/g) was lower than that reported by Al-perkhdri [23] in livers of camels (5.320 ppm) from central Kirkuk during spring and summer seasons. The concentration of Co in the liver of camels was higher than in sheep and goats. Unlike sheep and goats, camels eat more forage trees than grasses, which are generally richer in Co. This could be associated with the requirement of ruminants for essential elements such as Co for the synthesis of vitamin B_12_ [3].

Cd was present in the camel livers at a range of 0.314–0.774 µg/g. This result showed that the Cd concentrations were higher compared to camel livers from Morocco (0.25 mg/kg) and Nigeria (0.07–0.50 mg/kg) [12,22]. On the other hand, as reported by Al-perkhdri [23], in Kirkuk, the Cd concentration in the spring season in camel livers was 3.34 mg/kg, value that was higher than our result. In contrast, the concentration of Pb in camel liver in this study was at a higher level than Cd in camel liver of the breeds Wadha, Maghateer and Magaheem (0.0039, 0.0127 and 0.0091 mg/kg, respectively) in the eastern province of Saudi Arabia [24]. These differences may be due to exposure of the animals to heavy metals and high Cd concentrations in soil, forage and water (Figure 3, Figure 4 and Figure 5) during the spring season. The results of Cd concentrations in camels’ liver reared in central, eastern and southern districts (0.34, 0.35 and 0.31 µg/kg respectively) are below the values recommended by the European Commission (0.5 mg/kg) [27].

The accumulation of Cd in camels’ meat showed significant differences between districts through the spring season and was in the range of 0.134 to 0.358 µg/g. The Cd levels in the meat of camels from central and northern districts of Saudi Arabia were lower than those reported by Abdou and Mohamed [25] (0.07 mg/g) in camels from Kalubia Governorate, Egypt; Abdel Basset et al. [22] (0.12 mg/g) in camels from Morocco, Badis et al. [11] (0.08 mg/g) in camels from Algeria, and Bala et al. [12] (0.05 mg/kg) in camels from Nigeria. In contrast, in the current study the Cd concentration in camel meat was lower than the Cd concentration in meat from camels in Tabuk, Riyadh, Dammam, and Jizan (1.07, 1.02, 0.91, and 0.83 mg/g; respectively) in Saudi Arabia [26]. The result of this study was similar to that found by Khalafalla et al. [29] in Egypt in camels’ muscle, with Cd concentrations varying between 0.2 and 0.9 ppm. The mean Cd levels in camel meat in all districts was below the maximum levels allowed by the Codex Alimentarius Commission [30] and the European Commission (0.05 mg/kg wet weight) [27]. Cd concentrations in liver and meat increase in older animals and are related to Cd concentrations in the feed intake [31].

The increased concentration of Co in the meat of camels reared in the western district may be due to a higher level of Co in the feed intake as illustrated in Figure 3. The Co concentration in meat samples from the western district (3.255 µg/g) and other districts was lower than in meat of camels from central Kirkuk (8.19 ppm) and Mean areas in Iraq during spring and summer seasons were 4.203 ppm and 5.045 ppm respectively [23]. Several authors have reported the same Co concentration in camel meat compared to the concentration observed in the present study (Kadim et al. [21]: camels’ in Omani; ALI et al. [32]: camels in Pakistan; Asli et al. [33]: camel Iran). The presence of Co in the camel meat samples examined exceeded the maximum allowable limits (0.1 mg/kg fresh weight); the heavy metal concentration in muscle samples was generally lower than the maximum concentration accepted by the European Commission and Codex Alimentarius [27,30]. Even in small traces, heavy metals such as Cd, Pb and Co are very toxic elements that can cause various diseases, especially in children, as they are more susceptible to the effects of these metals [34]. The pastures are close to industrial areas with high levels of heavy metals, and heavy metals can enter the body through inhalation of dust, consumption of contaminated water or ingestion of contaminated feed [35]. 

There was no significant difference in Pb and Cd residues (*p* > 0.05) in whole blood, and rumen tissue between the samples tested from the central and eastern districts (Table 3). This indicates that heavy metals, especially Pb and Cd, can be absorbed with difficulty or only to a limited extent in the rumen. In contrast, Co showed high concentration in whole blood of camels from the central district, and in the rumen fluid and tissues of camels from the eastern district. Very limited data on heavy metals levels in whole blood, rumen fluid and rumen tissues have been reported in the literature, making it difficult to discuss the above results. Based on the previous study, we hypothesize that camels tolerate high concentrations of heavy metals via different mechanisms compared to other ruminants.

A significant negative correlation was observed between the levels of heavy metals (Pb and Cd) in rumen fluid and rumen tissue. This result explains that the concentration of Pb and Cd in the rumen fluid increased with the feed and water intake but the uptake from the rumen tissue was low. In contrast, no correlation was observed between meat, liver, and whole blood for the heavy metals Pb and Cd, and this result was contrary to that reported by Hussein et al. [24], who found a significant positive correlation between muscle and liver. In Saudi Arabia, a highly significant positive correlation was observed in muscle, serum and hair for Cd and Pb. It is known that these two metals (Ca and Pb) have no biochemical functions in animals, therefore they are considered toxic even at low concentrations in animal organs [36]. Finally, as observed, the accumulation of Pb and Cd in the muscle and liver of camels was lower. Muscular organs are the main edible part of the animal that can directly affect human health if they contain metals in excess of recommended standards. 

## 5. Conclusions

The districts show significant differences in the heavy metals tested, which can be explained by the level of pollution in each district. The rumen plays an important role in Pb, Cd and Co metabolism according to the significant correlation between rumen fluid and rumen tissue. The accumulation of Cd, Pb, and Co in meat and liver are below the recommended maximum limits, indicating that the risk of these metals for the health of consumers of camel meat is low. Further research using new techniques is strongly encouraged to study the metabolism and fermentation process in the rumen of these heavy metals, which are expected to be different from those of other ruminants.

## Figures and Tables

**Table 1:** The Pb, Cd and Co levels in liver (µg/g WW) of young camels reared in semi-arid conditions in five districts during spring.

District	Pb	Cd	Co
Central	2.981 ^a^	0.339 ^b^	4.621 ^a^
Eastern	1.670 ^b^	0.351 ^b^	1.262 ^c^
Western	0.906 ^c^	0.774 ^a^	2.461 ^b^
Southern	1.144 ^c^	0.314 ^b^	4.052 ^a^
Northern	1.621 ^b^	0.626 ^a^	1.118 ^c^
SEM	0.08	0.02	0.60
Significance	**	***	**

SEM = Standard error means; ^a,b,c^ = values within the column followed with different superscripts are significantly different ** = *p* < 0.01; *** = *p* < 0.001.

**Table 2:** The Pb, Cd and Co levels in meat (µg/g WW) of young camels reared in semi-arid conditions in five districts during spring.

District	Pb	Cd	Co
Central	1.386 ^b^	0.358 ^a^	1.705 ^b^
Eastern	1.001 ^d^	0.134 ^c^	1.396 ^c^
Western	1.229 ^b^	0.282 ^b^	3.255 ^a^
Southern	1.127 ^c^	0.193 ^c^	1.853 ^b^
Northern	2.058 ^a^	0.334 ^a^	2.664 ^a^
SEM	0.103	0.022	0.380
Significance	**	**	*

SEM = Standard error means; ^a,b,c,d^ = values within the column followed with different superscripts are significantly different * = *p* < 0.5; ** = *p* < 0.01.

**Table 3:** The Pb, Cd and Co levels in whole blood (µg/mL), rumen fluids (µg/mL WW) and rumen tissues (µg/mL WW) of young camels reared in semi-arid conditions in two districts during spring.

District	Pb	Cd	Co
Whole blood (µg/mL)			
Central	0.058	0.027	0.504
Eastern	0.061	0.027	0.038
SEM	0.005	0.001	0.099
Significance	NS	NS	**
Rumen fluids (µg/mL WW)			
Central	0.221	0.017	0.063
Eastern	0.202	0.022	0.110
SEM	0.018	0.001	0.016
Significance	NS	*	*
Rumen tissues (µg/g WW)			
Central	0.359	0.111	0.362
Eastern	0.407	0.129	0.518
SEM	0.016	0.004	0.120
Significance	NS	NS	*

SEM = Standard error means; the values within the column followed with different superscripts are significantly different * = *p* < 0.5; ** = *p* < 0.01; NS = Not significant.

**Table 4:** Correlation analysis of Pb mineral in different tissues in young camels during spring.

Organ	Meat	Liver	Whole Blood	Rumen Tissue	Rumen Fluid
Meat	1				
Liver	0.398 0.601	1			
Whole blood	0.555 0.445	−0.540 0.460	1		
Rumen tissue	−0.078 0.921	−0.656 0.343	0.478 0.521	1	
Rumen Fluid	−0.129 0.871	0.411 0.588	−0.439 0.560	−0.954 0.045 *	1

* = *p* < 0.5.

**Table 5:** Correlation analysis of Cd mineral in different tissues in camel during spring.

Organ	Meat	Liver	Whole Blood	Rumen Tissue	Rumen Fluid
Meat	1				
Liver	0.254 0.745	1			
Whole blood	−0.914 0.086	−0.329 0.671	1		
Rumen tissue	0.029 0.970	0.375 0.625	0.298 0.701	1	
Rumen Fluid	−0.211 0.789	−0.353 0.646	−0.141 0.858	−0.981 0.018 **	1

** = *p* < 0.01.

**Table 6:** Correlation analysis of Co mineral in different tissues in camel during spring.

Organ	Meat	Liver	Whole Blood	Rumen Tissue	Rumen FLUID
Meat	1				
Liver	−0.191 0.808	1			
Whole blood	0.328 0.671	0.496 0.503	1		
Rumen tissue	−0.307 0.692	−0.015 0.984	0.571 0.429	1	
Rumen Fluid	−0.640 0.359	0.653 0.347	−0.313 0.686	−0.362 0.638	1

## Data Availability

The data presented in this study are available on request from the corresponding author.
